# Single‐cell transcriptome analysis of human oocyte ageing

**DOI:** 10.1111/jcmm.16594

**Published:** 2021-05-26

**Authors:** Lihua Yuan, Ping Yin, Hua Yan, Xiufang Zhong, Chunxia Ren, Kai Li, Boon Chin Heng, Wuwen Zhang, Guoqing Tong

**Affiliations:** ^1^ Shuguang Clinical Medical College Shanghai University of Traditional Chinese Medicine Shanghai China; ^2^ Center of Reproductive Medicine Shuguang Hospital Affiliated to Shanghai University of Traditional Chinese Medicine Shanghai China; ^3^ Peking University Beijing China

**Keywords:** ageing, biological information data mining, gene expression, human oocyte, oocyte meiosis, sc​RNA‐seq, ubiquitination

## Abstract

Oocyte ageing is a key bottleneck and intractable challenge for in vitro fertilization treatment of aged female patients. The underlying molecular mechanisms of human oocyte ageing remain to be elucidated. Hence, this study aims to investigate the key genes and relevant biological signalling pathways involved in human oocyte ageing. We isolated mRNA for single‐cell RNA sequencing from MII human oocytes donated by patients undergoing intracytoplasmic sperm injection. Nine RNA‐seq datasets were analyzed, which included 6 older patients(average 42.67±2.25 years) and 3 younger patients (average 25.67±2.08 years). 481 differentially expressed genes (DEGs) were identified, including 322 upregulated genes enriched in transcription, ubiquitination, epigenetic regulation, and cellular processes, and 159 downregulated genes enriched in ubiquitination, cell cycle, signalling pathway, and DNA repair. The STRING database was used to analyse protein‐protein interactions, and the Cytoscape software was used to identify hub genes. From these DEGs, 17 hub genes were identified including 12 upregulated genes (UBE2C, UBC, CDC34, UBR1, KIF11, ASF1B, PRC1, ESPL1, GTSE1, EXO1, UBA1, KIF4A) and 5 downregulated genes (UBA52, UBE2V2, SKP1, CCNB1, MAD2L1). The significant key biological processes that are associated with these hub genes include ubiquitin‐mediated proteolysis, ubiquitination‐related pathways, oocyte meiosis, and cell cycle. Among these, UBE2C may play a crucial role in human oocyte ageing.

## 1 INTRODUCTION

Pregnancy rate decreases sharply for female adults from 37 years of age onwards.^1^ The accelerated decrease in the number and quality of oocytes are the key factors affecting fertility in older women. Compared with young women, the IVF pregnancy rate in older infertile women is significantly reduced (<5%), and the probability of aneuploidy in MII oocytes is significantly increased.^2^ Oocyte ageing is characterized by deterioration of a series of molecular processes during ageing, which negatively affect fertilization and development, such as diminished fertilization rate, aberrant fertilization, and embryonic chromosomal abnormalities.^3^ Oocyte quality can be defined as the oocyte's ability to complete meiosis and provide a complete complement of RNA, protein, and energy molecules needed to support early embryogenesis before embryonic genome activation.^4^ Although female patient age is highly correlated with oocyte quality and pregnancy outcome, the molecular mechanisms of oocyte ageing have not yet been rigorously characterized, and there needs to be a better understanding of this ageing process in order to develop new therapeutic strategies for older women undergoing IVF treatment.

The regulation of transcriptional activity within oocytes determines their developmental potential.^5^ Microarray technology has made high‐throughput transcriptomics a reality. A previous study on full genome microarray analysis of MII oocyte has demonstrated considerable differences in the gene transcriptional levels of mature MII oocytes between young and old patients, with differentially expressed genes (DEGs) being identified in various aspects of cell cycle regulation, chromosome alignment, sister chromatid separation, oxidative stress, and protein ubiquitination.^6^

Compared to microarray analysis, single‐cell transcriptome sequencing (scRNA‐seq) studies present the overall gene expression of a single cell and are particularly better at detecting transcription of unknown genes at a more precise and accurate level, which can quantitatively reveal gene expression defects caused by age‐associated changes in gene regulation and epigenetic variation. scRNA‐seq has been widely used in sequencing studies of gene expression during human oocyte maturation. For example, human oocytes maturing in vitro and in vivo have been analysed by RNA‐seq, and several key genes and cell signalling pathways that regulate human oocyte maturation from the metabolic perspective have been identified.^7^ A recent study had identified 357 DEGs between old and young human oocytes via RNA‐seq, which were mainly associated with transcriptional activation, oxidative stress, immune function, and catalytic activity.^8^ Thus, scRNA‐seq cannot only help us study how genes are expressed and regulated, but can also facilitate identification of relevant functional genes in aged oocytes. Furthermore, combined with bioinformatics data mining tools, it is possible to find other key signalling pathways that are associated with oocyte ageing.

Therefore, we investigated the ageing‐associated gene expression signatures of MII oocytes by scRNA‐seq. Subsequently, we analysed and processed the sequencing data to screen DEGs, enriched the functional and signalling pathways of DEGs, built protein interaction networks, and screened the key genes and important signalling pathways in the network. The results of this study could provide the theoretical basis for future research on the molecular mechanisms of oocyte ageing from a different perspective.

## 2 MATERIALS AND METHODS

### 2.1 Human oocyte samples

All research participants were patients undergoing intracytoplasmic sperm injection (ICSI) treatment in the past two years at the reproductive medicine centre of Shuguang hospital affiliated to Shanghai university of Traditional Chinese medicine. Oocytes were donated by ICSI patients who had signed consent forms. This study was approved by the Ethics Committee of Shuguang University of Traditional Chinese Medicine (Shanghai, China). There were two age groups. A total of 12 MII oocytes were analysed including 6 older (age ≥40, average 42.67 ± 2.25 years) and 6 younger (age <30, average 26.83 ± 1.94 years) unfertilized MII oocytes.

### 2.2 Controlled ovarian stimulation and oocyte retrieval

All included patients received standard antagonist protocol for ovarian stimulation. Briefly, patients were injected with recombinant FSH (Gonal‐F, Merck, Italy) on the 2nd day of their menstrual cycle with a starting dose of 150 IU/d. Transvaginal ultrasound scanning and blood hormone sampling were applied to monitor follicle growth. When more than two follicles grew larger than 18mm in diameter, 0.1mg of gonadotropin‐releasing hormone agonist (Triptorelin, Ferring GmbH, Germany) was injected subcutaneously and 4000 IU of human chorionic gonadotropin was injected intramuscularly to trigger final maturation. After 36 hours post‐triggering, transvaginal oocyte retrieval was performed under the guidance of ultrasound scanning with anaesthesia.

### 2.3 RNA extraction

Oocytes were exposed to a solution with 80 IU hyaluronidase (Sigma) and manipulated with pipettes to remove cumulus cells. Then, the oocyte was rinsed in Ca^2+^/Mg^2+^‐free PBS solution and individually placed in 0.2 ml PCR tubes containing 10 μl of TRIzol reagent (Invitrogen). The samples were stored at −80℃ pending further processing, until RNA was isolated with the EZNA^®^ Total RNA Kit Ⅱ (OMEGA).

### 2.4 Library construction and RNA sequencing analysis

RNA extracted from single oocytes was amplified by Smart‐seq2.^9^ Briefly, poly (A) mRNA was isolated from total cellular RNA using oligo (dT) magnetic beads; a fragmenting buffer was added to break the mRNA into short fragments. Using the segmented mRNA as template, the first cDNA strand was synthesized by adding six‐base random primers, and then the second cDNA strand was synthesized by adding a mixture of buffer, dNTPs, RNase H, and DNA polymerase I. Double‐stranded cDNA was purified using AMPure XP beads (Beckman Coulter). The purified double‐stranded cDNA was repaired at the end, followed by the addition of a poly A tail and ligation of the sequencing connector. Then, AMPure XP beads were used for fragment size selection. Finally, PCR amplification was performed to obtain the final cDNA library. The libraries were sequenced by an Illumina HiSeq X‐ten platform with 150 bp paired‐ends. The sequencing data that met the requirements were subjected to further removal of low‐quality sequences and joint sequences to obtain clean reads. The data filtering standards are as follows: Remove Reads connector sequence; Remove the parts with average quality of less than Q20 from Reads 3' end to 5' end (5 bp window); Remove sequences of final length less than 50 bp; No uncertain bases in the sequence. After the original data passed the quality filtering, the quality control analysis of the quality filtering was carried out by the FastQC software (version0.11.3) (Figure S1). Then, Reads were mapped to the human genome GRCh38 using the STAR software by default settings (version2.5.3α).^10^ FeatureCount statistics were used to compare the values to the genes, followed by analysis of differentially expressed genes (DEGs) by the DESeq2 software (version 1.20.0).^11^ The genes with |log2foldchange|≥1 and Q‐value<0.05 were considered as differentially expressed genes.

### 2.5 Genetic ontology and pathway enrichment analysis of DEGs

DEGs were divided into upregulated genes and downregulated genes for statistical analysis. Enrichment analysis of genetic ontology (GO) on screened DEGs was performed using the DAVID online analysis website (Version 6.8, https://david.ncifcrf.gov/). GO enrichment analysis includes molecular function (MF), biological process (BP), and cellular component (CC). KEGG pathway was performed by the clusterProfiler R package (Version 3.8). GO terms and KEGG pathway with a *P* value of <.05 were considered to be statistically significant.

### 2.6 PPI network and identification of Hub genes

The search tool for the retrieval of interacting genes/proteins (STRING) (Version 11.0, http://string‐db.org/) was used to analyse protein‐protein interactions (PPI). We built up protein interaction networks of DEGs. In order to make the results more reliable, data with interaction score >0.4 were selected from protein‐protein interactions to construct a PPI network. The Cytoscape software (Version 3.7.2, http://www.cytoscape.org/) was used to visualize and analyse the STRING‐based DEGs interaction network. We deleted discrete nodes and edges. The Cytoscape plug‐in tool MCODE was used to analyse the PPI network module to obtain the DEGs network hotspot module. MCODE score >4 and the number of nodes >5 were selected. The filtering parameters are as follows: degree cut‐off>2, Node score cut‐off>2, K‐core>2, and Max depth = 100. The cytoHubba plug‐in of the Cytoscape software is a plug‐in specifically used to identify key targets and sub‐networks of complex networks. The top 30 genes with high degrees and with MCODE score ≥10 were utilized as the cut‐off criterion.^12^ Then, the Cytoscape software was used to visualize and analyse the STRING‐based hub genes interaction network. Finally, the GO and KEGG pathway were analysed by DAVID.

### 2.7 Statistical analyses

All statistical analyses of scRNA‐seq data were performed using the R software, DAVID website, STRING, and Cytoscape software. Visualization of data was made using R packages DESeq2, ggplot2 and GraphPad Prism 8.0. In the identification of DEGs, adjusted *P* value <.05 was considered statistically significant. For foundational enrichment analysis, *P* value <.05 was considered statistically significant. IBM SPSS Statistics V.22.0 was used to analyse the clinical data. Continuous quantitative data were analysed using the t test. Data showing normal distribution were presented as mean ± SD.

## 3 RESULTS

### 3.1 Gene expression profile data

**FIGURE 1:**
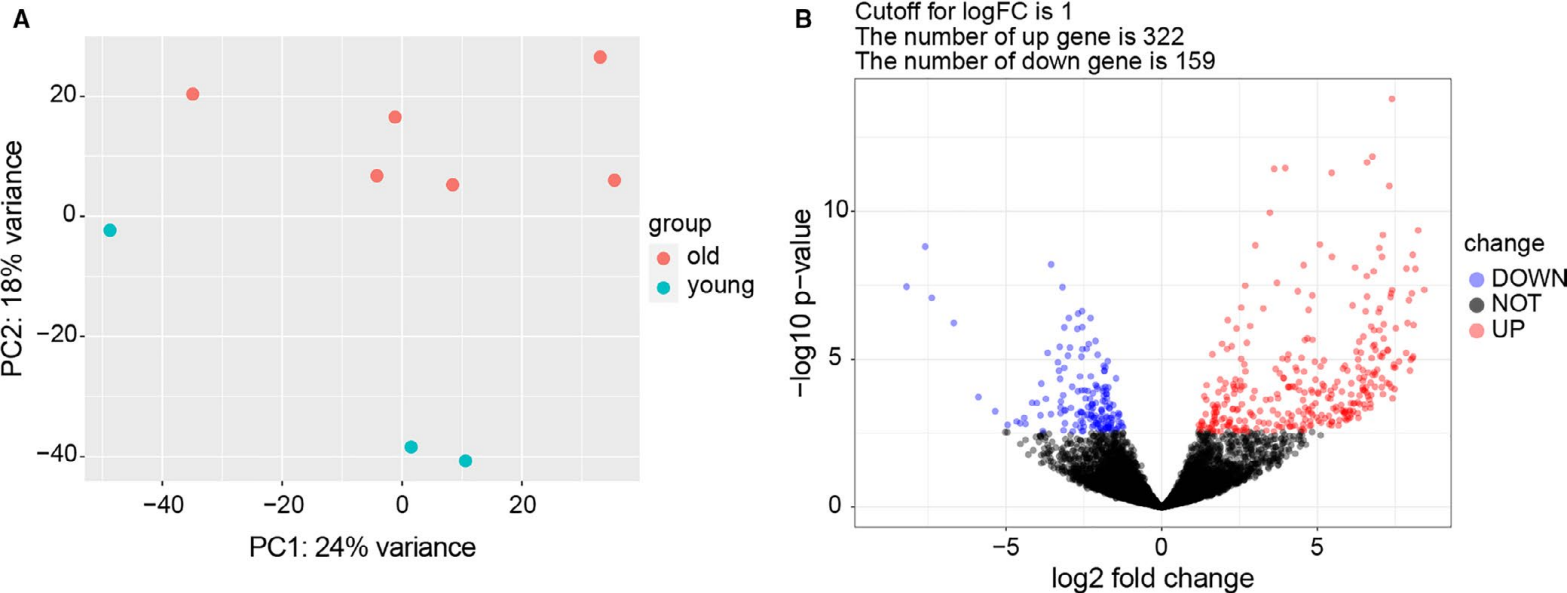
scRNA‐seq of old and young human oocytes. A, Principal component analysis (PCA) score chart in 6 old and 3 young samples, showing that there was a tendency to separate populations. B, A total of 481 DEGs were identified in age group ≥40 vs. <30. DEGs between oocytes of older and younger women are shown in the volcano map; the *X* axis represents the log2 fold change and the *Y* axis displays the −log10(*P* value); genes that were upregulated in older female oocytes are shown in red, downregulated are shown in blue, and black dots represent genes with no significant difference in expression level

We performed RNA‐seq analysis on 6 old and 6 young unfertilized MII oocytes to study gene expression changes during oocyte ageing. Based on the results of scRNAseq, oocytes exhibited differential gene expression in the age group <30 (average 25.67 ± 2.08 years) versus ≥40(average 42.67 ± 2.25 years). Samples from the two groups were further analysed by principal component analysis (PCA), which was mainly used to characterize the trend of inter‐group separation in the experimental model, to ascertain whether there were abnormal data points, and to determine the degree of variation between groups and within groups from the original data. Principal component analysis (PCA) in 6 older and 6 younger oocyte samples showed that three of the younger samples had no tendency to separate from the older samples (Figure S2). Then, the clinical data of the two groups were reviewed. There were significant differences in basal follicle‐stimulating hormone (bFSH), MⅡ oocyte transition rate, and available embryo rate between the three young oocytes that were excluded and the three young oocytes that were included in the analysis (Tables S1 and S2). Hence, we eliminated these three younger samples. PCA showed that the data of the three included oocyte samples of younger women were clustered apart from those of older woman (Figure 1A). The DESeq2 package in R language was used to screen the DEGs. Of these, 481 DEGs were screened, including 322 upregulated genes and 159 downregulated genes in the older versus younger oocytes. Transcriptome changes can be observed in volcano plots presented in Figure 1B.

### 3.2 Enrichment analysis

In order to evaluate the characteristics of aged oocytes and determine which major functional categories are affected by age, we next analysed the biological functions by using Gene Ontology (GO) and KEGG pathway analysis of differentially expressed genes (DEGs) with the DAVID website. In comparison with the younger oocytes, fifty‐one GO biological processes were identified at the 0.05 significance level to be under‐ or overrepresented in the older oocytes. The categorized genes are shown in Table 1 and Table 2.

**TABLE 1:** Functional enrichment of upregulated genes that are differentially expressed in older oocytes versus younger oocytes

Category	Term	*P* value	Count
Transcription	Transcription from RNA polymerase II promoter	.003	18
	Positive regulation of transcription from RNA polymerase II promoter	.006	27
	Negative regulation of transcription from RNA polymerase II promoter	.035	19
	RNA splicing	.016	8
Ubiquitination	Protein K48‐linked ubiquitination	.006	5
	Protein ubiquitination	.011	13
	Ubiquitin‐dependent protein catabolic processes	.025	8
	Proteasome‐mediated ubiquitin‐dependent protein catabolic processes	.041	8
Epigenetic regulation	DNA methylation	3.73E‐05	6
	Histone H3‐K4 demethylation	.005	3
	Histone H2A acetylation	.022	3
	Regulation of gene expression by genetic imprinting	.025	3
Cell proliferation	Regulation of cell proliferation	.009	9
	Negative regulation of cell growth	.042	6
DNA damage response	Cellular response to DNA damage stimulus	.017	9
Protein transport	Vesicle‐mediated transport	.033	7
Cytoskeleton	Cytoskeleton organization	.041	7
Cell junction	Cell‐cell junction organization	.031	3
Signalling pathway	Regulation of smoothened signalling pathway	.035	3
Metabolism	Regulation of fatty acid transport	.031	2

**TABLE 2:** Functional enrichment of downregulated genes that are differentially expressed in older oocytes versus younger oocytes

Category	Term	*P* value	Count
Ubiquitination and cell cycle	Positive regulation of ubiquitin‐protein ligase activity involved in regulation of mitotic cell cycle transition	1.47E‐04	6
	G2/M transition of mitotic cell cycle	2.97E‐04	7
	Negative regulation of ubiquitin‐protein ligase activity involved in mitotic cell cycle	.001	5
	Proteasome‐mediated ubiquitin‐dependent protein catabolic processes	4.00E‐04	8
	Anaphase‐promoting complex‐dependent catabolic processes	.002	5
Ubiquitination	Protein polyubiquitination	2.20E‐04	8
	Protein ubiquitination involved in ubiquitin‐dependent protein catabolic processes	.003	6
	Positive regulation of proteasomal ubiquitin‐dependent Protein catabolic processes	.009	4
Signalling pathway	NIK/NF‐kappaB signalling	.010	4
	MAPK cascade	.030	6
	Fc‐epsilon receptor signalling pathway	.030	5
	Stimulatory C‐type lectin receptor signalling pathway	.032	4
DNA repair	Global genome nucleotide‐excision repair	.020	6
	DNA repair	.019	3
Apoptosis	Negative regulation of apoptotic processes	.031	8
Translation	Translational initiation	.013	5
Protein targeting	SRP‐dependent co‐translational protein targeting to membrane	.024	4
Regulation of mRNA	Regulation of mRNA stability	.031	4
Energy	ATP synthesis coupled electron transport	.033	2
Vasoconstriction	Vascular smooth muscle contraction	.026	2

**FIGURE 2:**
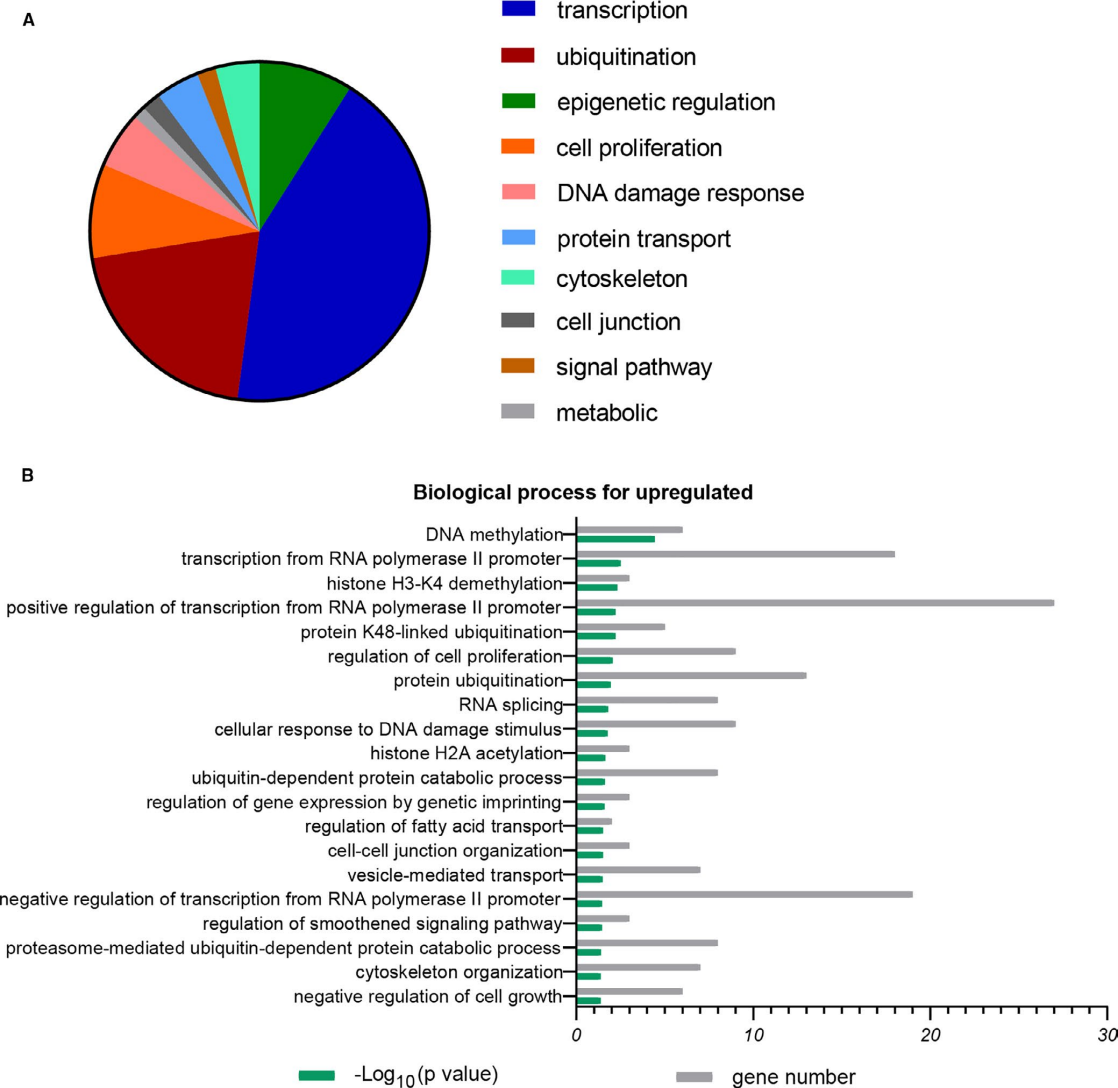
Biological processes associated with upregulated differentially expressed genes (DEGs) in older oocytes versus younger oocytes. A, The GO annotation for biological processes of upregulated genes was allocated into 10 classes. B, Representative biological processes for DEGs. *X* axis represents percentage of genes or −log_10_(*P* value); *Y* axis represents molecular functions or biological processes, *P* < .05

**FIGURE 3:**
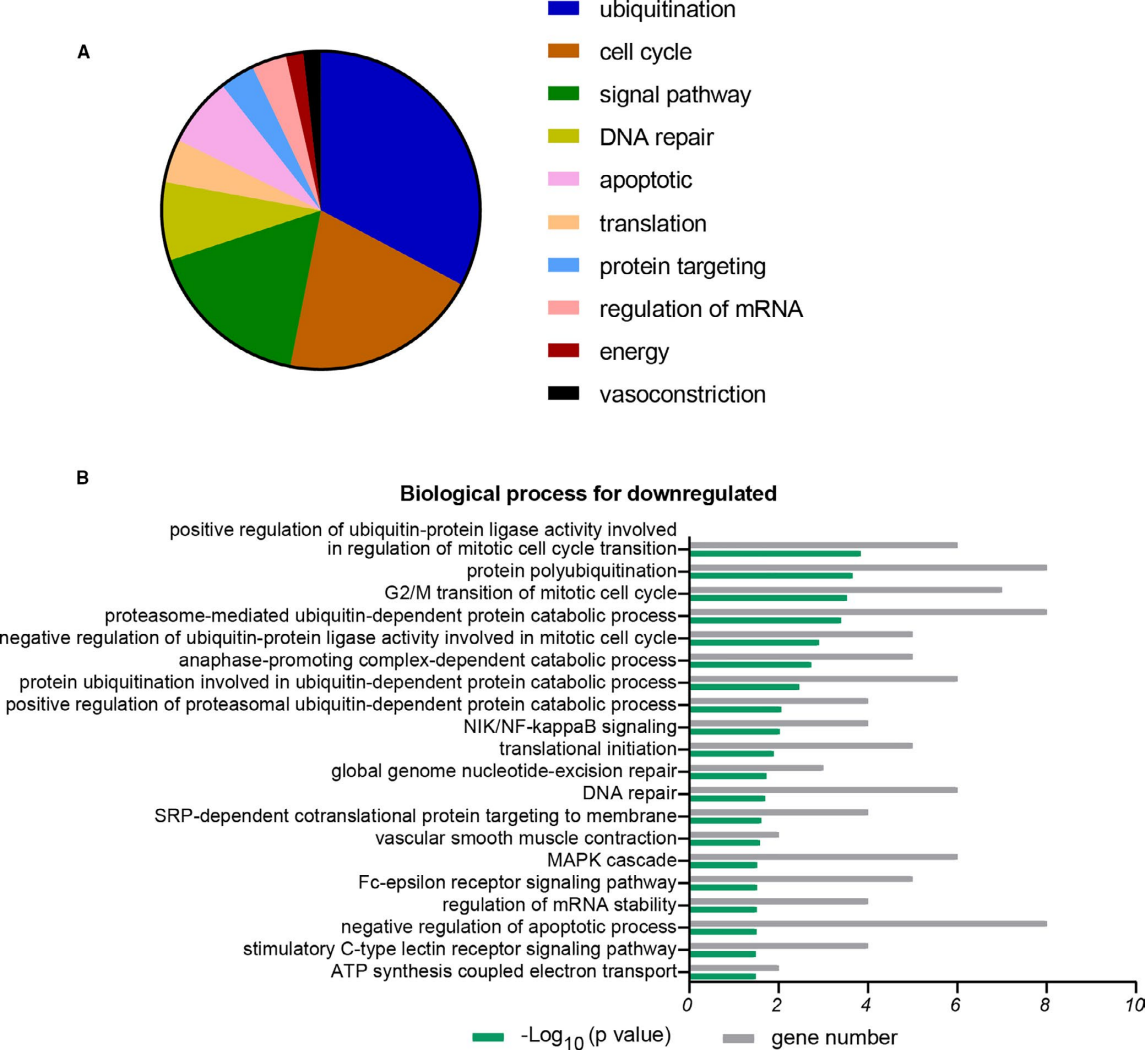
Biological processes associated with downregulated DEGs in older oocytes versus younger oocytes. A, The GO annotation for biological processes of downregulated genes were allocated into 10 classes. B, Representative biological processes for downregulated genes. *X* axis represents percentage of genes or −log_10_(P value); *Y* axis represents molecular functions or biological processes. *P* < .05

The top 20 terms of biological process (BP) for upregulated DEG are shown in Figure 2 and Table 1. The dominance of the categories was transcription (43%), ubiquitination (20%), epigenetic regulation (9%), and cellular processes (9%) (Figure 2A). In the transcription category, the main enrichment terms were transcription from RNA polymerase II promoter (*P* = .003), positive/negative regulation of transcription from RNA polymerase II promoter (*P* = .006, 0.035), and RNA splicing (*P* = .016). Regulation of transcription determines oocyte development.^13^ RNA polymerases bind to specific promoter regions, thereby initiating RNA strand elongation. Positive or negative regulation of transcription from the RNA polymerase II promoter may explain the reduced potential of older oocytes. In addition, other major ubiquitination terms were highly overrepresented, such as protein K48‐linked ubiquitination (*P* = .006) and protein ubiquitination (*P* = .011). However, the BP for downregulated DEGs was mainly in the processes: ubiquitination (33%), cell cycle (20%), signalling pathway (17%), and DNA repair (8%) (Figure 3; Table 2). The category of ubiquitination not only contained the function of protein polyubiquitination (*P* = 2.20E‐04), but also ubiquitination‐related cell cycle and ubiquitin‐protein catabolic processes, such as positive regulation of ubiquitin‐protein ligase activity involved in regulation of mitotic cell cycle transition (*P* = 1.47E‐04), negative regulation of ubiquitin‐protein ligase activity involved in mitotic cell cycle (*P* = .001), proteasome‐mediated ubiquitin‐dependent protein catabolic processes (*P* = 4.00E‐04), protein ubiquitination involved in ubiquitin‐dependent protein catabolic processes (*P* = .003), and positive regulation of proteasomal ubiquitin‐dependent protein catabolic processes (*P* = .009).

**FIGURE 4:**
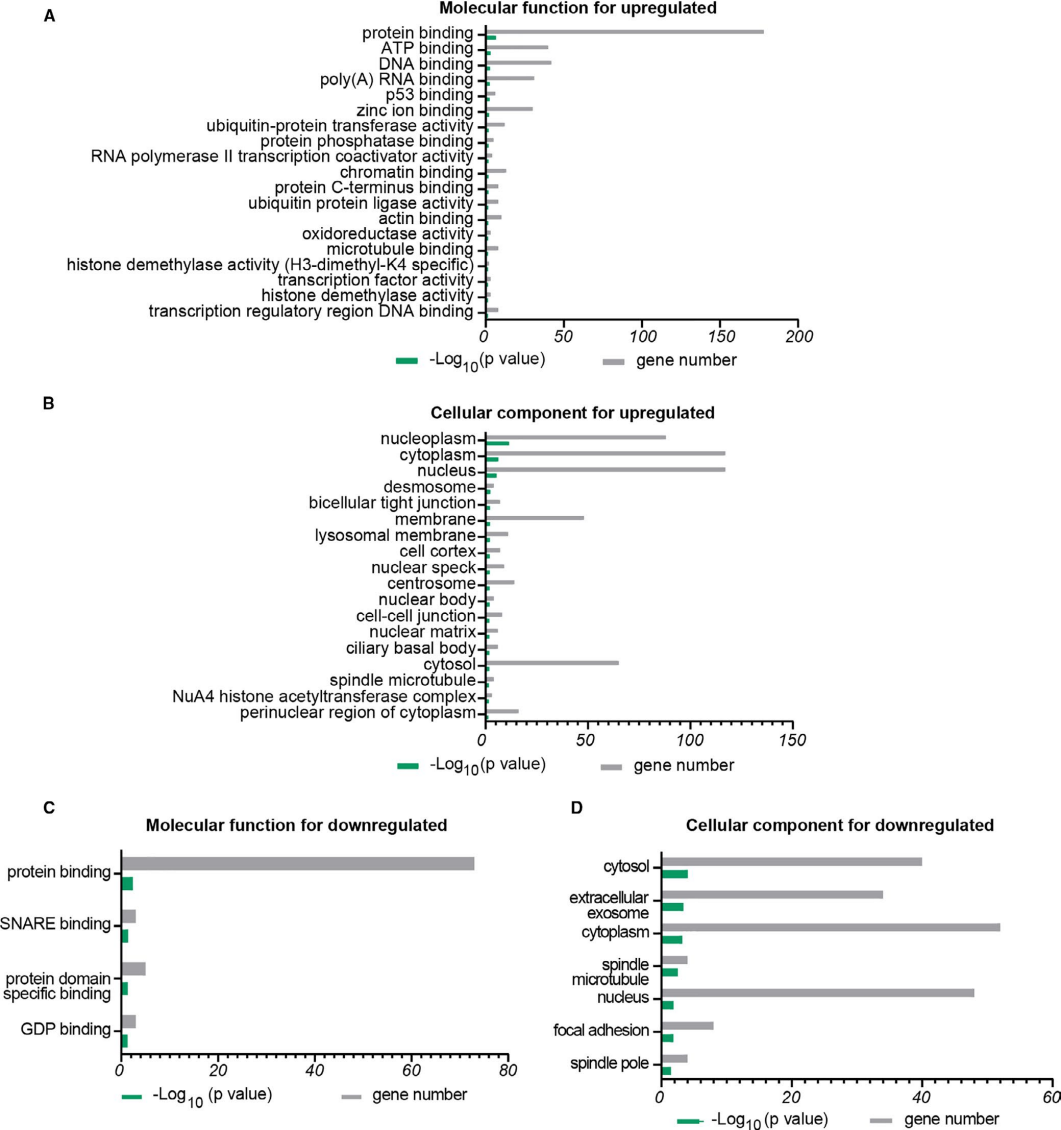
Molecular functions and cellular components associated with DEGs in older oocytes versus younger oocytes. A, Molecular functions of upregulated DEGs in older oocytes versus younger oocytes. B, Cellular components associated with upregulated DEGs in older oocytes versus younger oocytes. C, Molecular functions of downregulated DEGs in older oocytes versus younger oocytes. D, Cellular components and biological processes associated with upregulated DEGs in older oocytes versus younger oocytes. *X* axis represents percentage of genes or −log_10_(P value); *Y* axis represents molecular functions or biological processes, *P* < .05

With regard to molecular function (MF), upregulated DEGs were mainly enriched in binding and activity. ‘Binding’ mainly represented protein binding (*P* = 3.06E‐07), ATP binding (*P* = 9.73E‐04), DNA binding (*P* = .002), poly(A)RNA binding (*P* =.003), chromatin binding (*P* = .020), and microtubule binding (*P* = .044). The following sub‐processes under ‘catalytic activity’ were enriched: ubiquitin‐transferase activity (*P* = .027), ubiquitin‐protein ligase activity (*P* = .015), and histone demethylase activity (*P* = .049). In addition, RNA polymerase II transcription coactivator activity (*P* = .049) and transcription factor activity (*P* = .050) were enriched in ‘transcriptional activity’ (Figure 4A). The MF analysis showed that ‘binding’ and ‘activity’ reflect the changes of GO term of BP. The cellular component (CC) analysis for upregulated DEGs was mainly enriched in nucleoplasm, cytoplasm, and membrane. The CC analysis showed that most of the transcripts represent genes involved in intracellular processes (Figure 4B). By contrast to the MF analysis, downregulated DEGs were also enriched in ‘binding’, which included protein binding (*P* = .004), SNARE binding (*P* = .037), protein domain specific binding (*P* = .048), and GDP binding (*P* = .049) (Figure 4C). Additionally, the CC analysis for downregulated DEGs was mainly enriched in cytosol, cytoplasm, nucleus, and extracellular exosome, thus implying that downrepresented DEGs included intra‐ and extracellular processes (Figure 4D).

**FIGURE 5:**
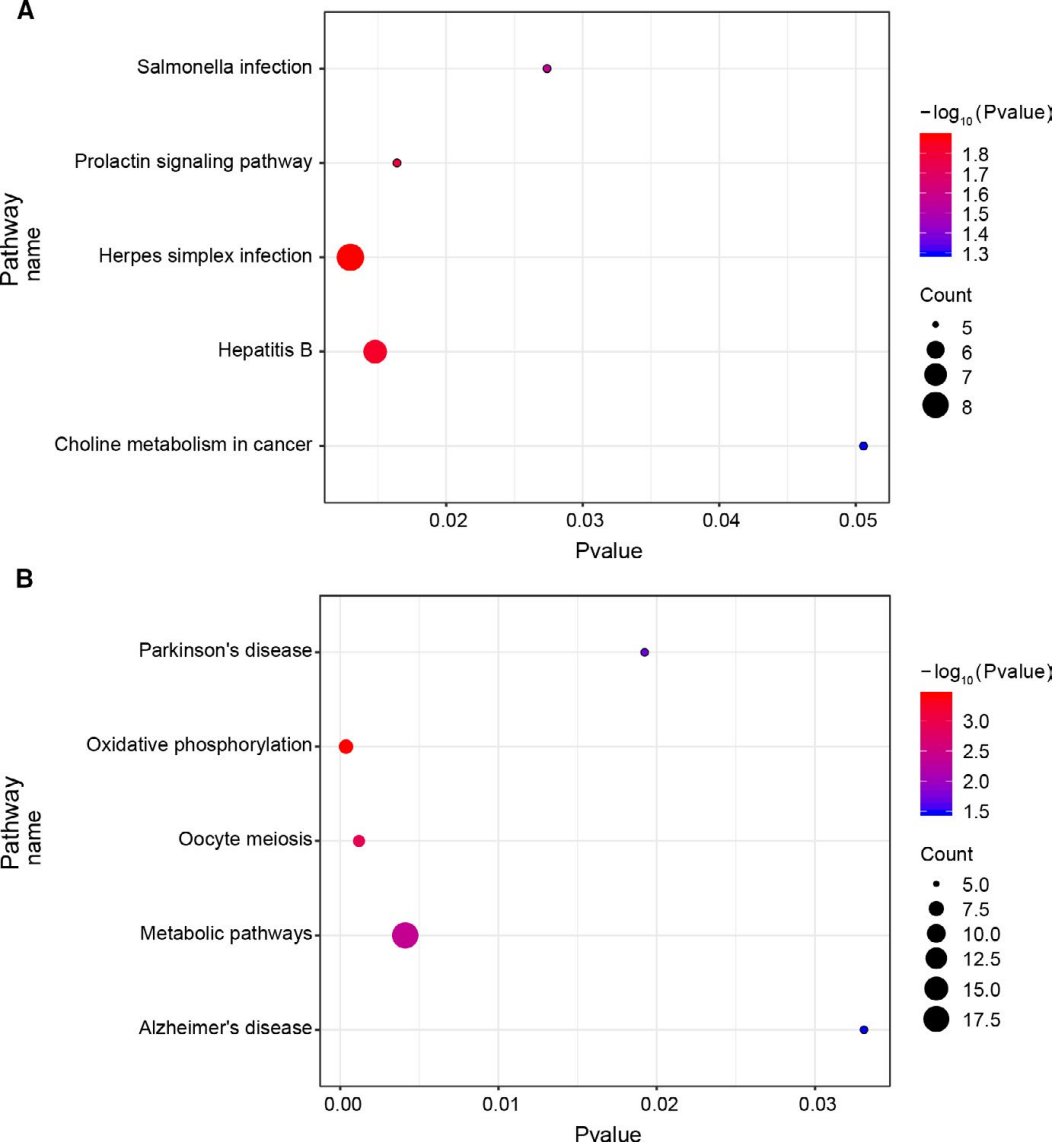
KEGG analysis for upregulated (A) and downregulated DEGs (B) in older oocytes vs younger oocytes. *P* < .05

The KEGG pathway of upregulated DEGs was mainly enriched in infections, prolactin, and cancer related pathways. The KEGG pathway of downregulated DEGs includes oxidative phosphorylation and oocyte meiosis (Figure 5).

### 3.3 PPI network construction and hot module analysis

**FIGURE 6:**
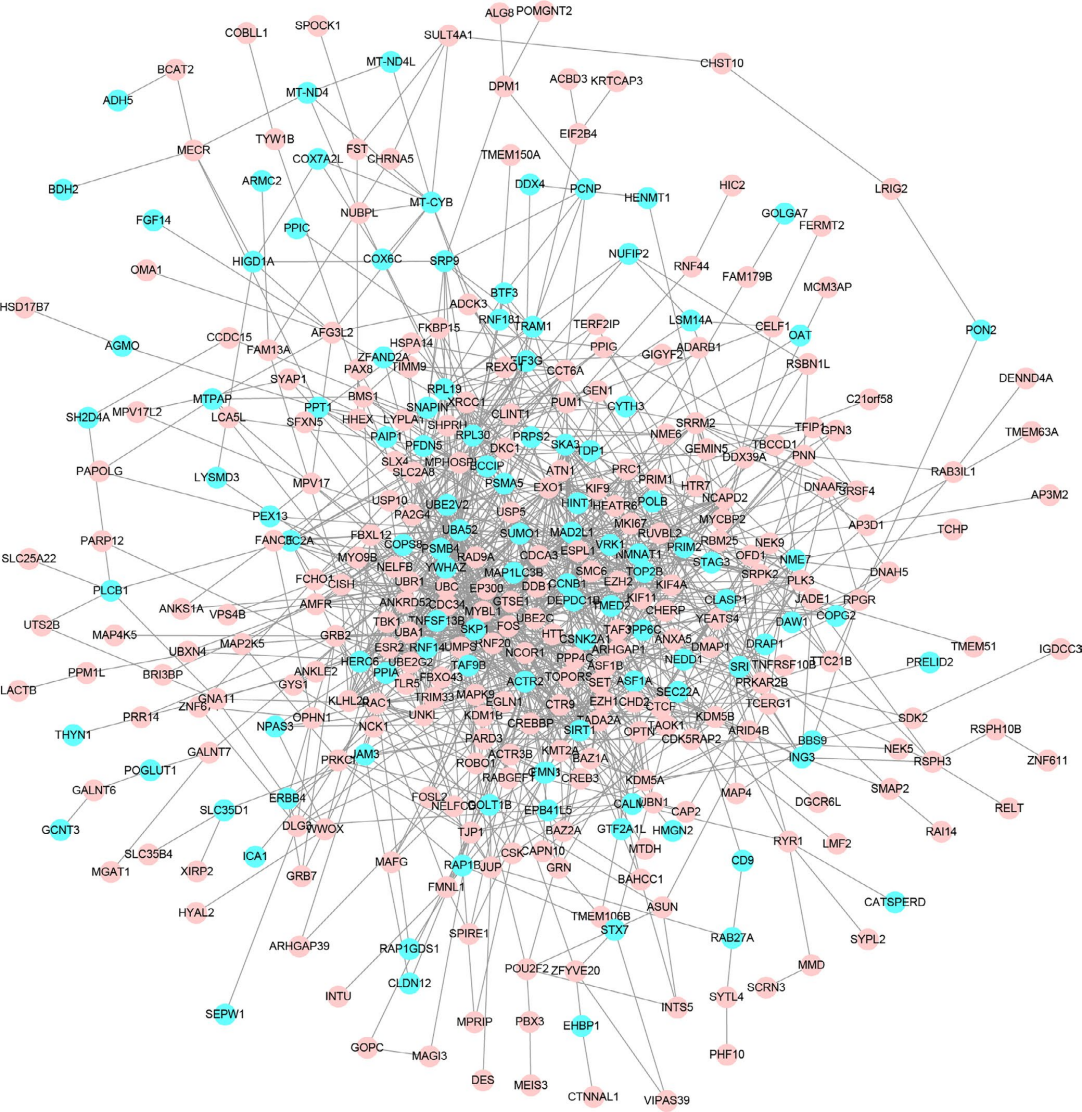
The PPI network of DEGs were constructed by Cytoscape, including 358 nodes and 1280 edges (discrete nodes and edges were deleted). Circles represent genes, and lines represent protein interactions between genes. Upregulated DEGs were marked in pink; downregulated DEGs were marked in blue. Data with interaction score >0.4 were selected from protein‐protein interactions to construct a PPI network

**FIGURE 7:**
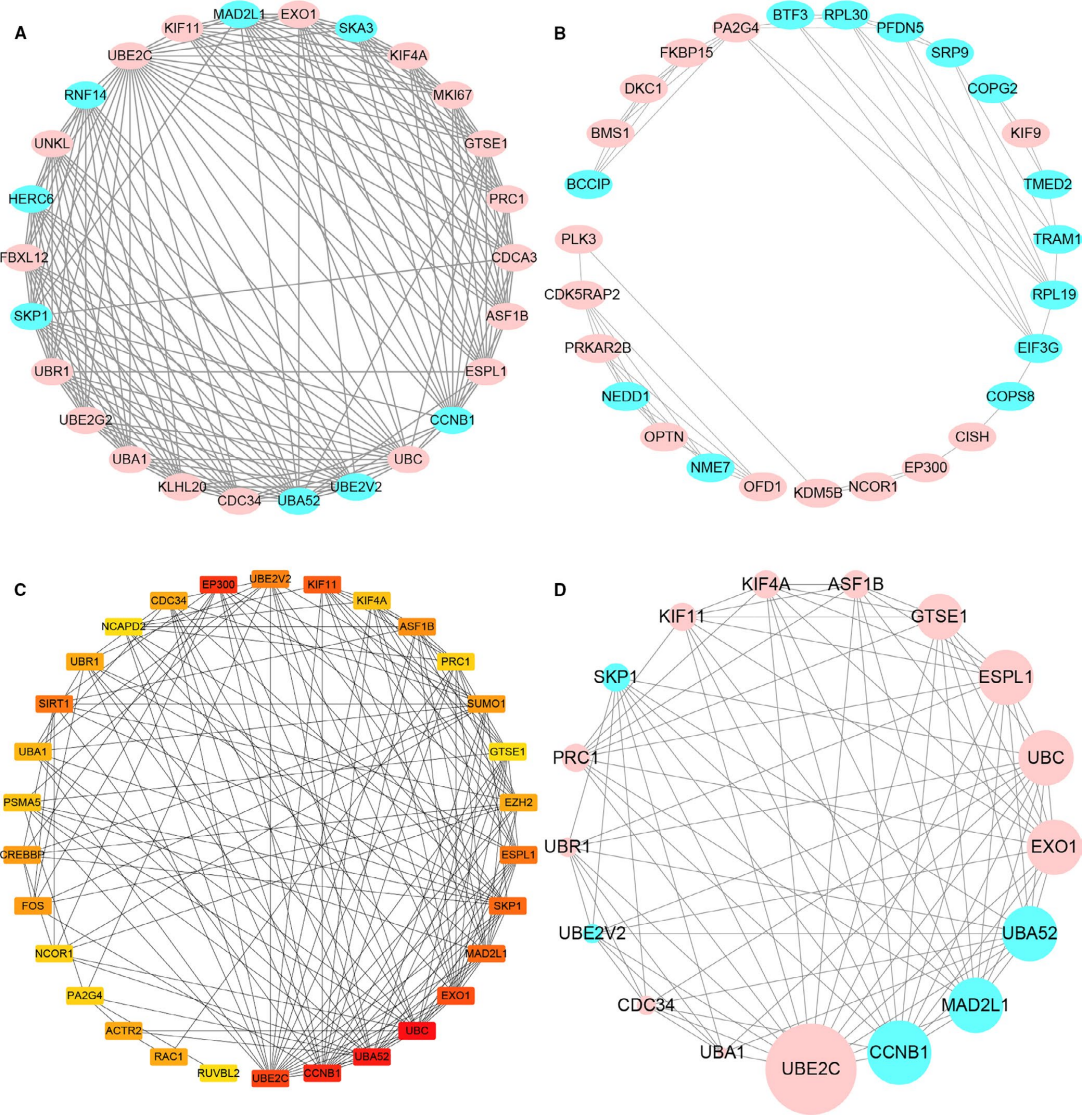
Establishment of modules and identification of hub genes for oocyte ageing. A & B, Two significant modules identified from the PPI network using the molecular complex detection method (MCODE) with a score of >4.0 and nodes >5. A: Module 1, MCODE score = 14.72; B: Module 2, MCODE score = 4.23. C, The top 30 DEGs in the interrelationship network analysed by degree in cytoHubba, the higher the rank, the redder the colour. D, PPI network of the hub genes. The circle size indicates the degree of association of the gene in the network; upregulated genes are marked in pink; downregulated genes are marked in blue

The PPI network of DEGs was constructed at the protein level using the STRING online tool. Based on the search results of the STRING database, we constructed a protein interaction network of DEGs by the Cytoscape software, as shown in Figure 6. The whole network has 358 nodes and 1280 edges. The plug‐ins MCODE in Cytoscape were used to perform module analysis. In total, two modules (modules 1 and 2) with score >4 and nodes >5 were detected by MCODE. Module 1 covers 20 nodes and 184 edges, with a score of 14.72, including 18 upregulated genes and 8 downregulated genes (Figure 7A). Functional enrichment analysis of these genes showed that the most significant top 5 enrichment results in GO function BP were mainly concentrated in ubiquitination‐related functions, which demonstrated dysregulation of ubiquitination pathways in older oocytes. Module 2 covers 27 nodes and 55 edges, with a score of 4.23, containing 14 upregulated genes and 13 downregulated genes (Figure 7B). The genes for Module 2 are mainly enriched in mitosis, cell cycle, rRNA processing, translation and protein localization functions.

### 3.4 Hub gene screening

The STRING database with Cytoscape software and cytoHubba plug‐in was combined to screen key genes that may be related to oocyte ageing. The top 30 genes with the highest degrees of connectivity (Figure 7C) and the genes with MCODE scores ≥10 (Figure 7A) were identified hub genes. Finally, a total of 17 hub genes were screened including 12 upregulated DEGs and 5 downregulated DEGs (Figure 7D, Table 3). The upregulated DEGs included UBE2C (|log2FC| = 7.86), UBC (|log2FC| = 3.91), CDC34 (|log2FC| = 2.32), UBR1 (|log2FC| = 5.08), KIF11 (|log2FC| = 3.05), ASF1B (|log2FC| = 3.36), PRC1 (|log2FC| = 1.63), ESPL1 (|log2FC| = 4.94), GTSE1 (|log2FC| = 2.01), EXO1 (|log2FC| = 4.20), UBA1 (|log2FC| = 2.62) and KIF4A (|log2FC| = 1.49); the downregulated DEGs including UBA52 (|log2FC| = 1.38), UBE2V2 (|log2FC| = 2.23), SKP1 (|log2FC| = 2.34), CCNB1 (|log2FC| = 1.84), and MAD2L1 (|log2FC| = 1.62). The top 10 biological process for the hub genes was mainly enriched in the term of ‘ubiquitination’, ‘mitotic cell cycle’, ‘DNA damage response, signal transduction by p53 class mediator resulting in cell cycle arrest’, and ‘stress‐activated MAPK cascade’ (Table 4). Then, the KEGG pathway analysis of hub gene was performed by DAVID, and three pathways were found to be significantly enriched, which included ‘oocyte meiosis’, ‘cell cycle’, and ‘Ubiquitin‐mediated proteolysis’ (Table 5). Hence, it can be inferred that both the degree of UBE2C and fold were dominant. Therefore, UBE2C may play a crucial role in the PPI network of hub genes.

**TABLE 3:** Identification of hub genes by degree and MCODE score of the Cytoscape software

Cytoscape‐cytoHubba		Cytoscape‐MCODE		Degree & MCODE score
Name	Degree	Name	MCODE score	Name
UBC	60	UBC	13	UBE2C
UBA52	59	UBA52	13	CCNB1
CCNB1	42	UBE2C	13	UBA52
EP300	37	SKP1	13	UBC
UBE2C	34	UBE2V2	13	MAD2L1
EXO1	33	UBE2G2	13	EXO1
KIF11	31	CDC34	13	SKP1
SKP1	29	RNF14	13	ESPL1
MAD2L1	29	KLHL20	13	GTSE1
SIRT1	27	FBXL12	13	PRC1
ESPL1	27	HERC6	13	ASF1B
UBE2V2	26	UBA1	13	KIF4A
ASF1B	25	UNKL	13	KIF11
FOS	23	UBR1	13	UBE2V2
CREBBP	23	SKA3	11	CDC34
SUMO1	23	GTSE1	11	UBR1
UBR1	22	MAD2L1	11	UBA1
RAC1	22	MKI67	11	
EZH2	22	CCNB1	11	
ACTR2	22	ESPL1	11	
CDC34	22	ASF1B	11	
UBA1	21	PRC1	11	
KIF44	20	KIF11	11	
PSMA5	20	KIF4A	11	
PA2G4	19			
NCOR1	19			
PRC1	19			
NCAPD2	18			
GTSE1	18			
RUVBL2	18			

**TABLE 4:** The top 10 biological process associated with the hub genes

Term	*P* value	Count	Genes
Positive regulation of ubiquitin‐protein ligase activity involved in regulation of mitotic cell cycle transition	6.98E‐09	6	UBA52, SKP1, CCNB1, UBC, MAD2L1, UBE2C
Proteasome‐mediated ubiquitin‐dependent protein catabolic processes	2.10E‐08	7	GTSE1, UBA52, CDC34, SKP1, UBC, MAD2L1, UBE2C
Negative regulation of ubiquitin‐protein ligase activity involved in mitotic cell cycle	5.14E‐07	5	UBA52, CCNB1, UBC, MAD2L1, UBE2C
Anaphase‐promoting complex‐dependent catabolic processes	7.91E‐07	5	UBA52, CCNB1, UBC, MAD2L1, UBE2C
Protein ubiquitination involved in ubiquitin‐dependent protein catabolic processes	1.11E‐05	5	UBA52, CCNB1, UBC, MAD2L1, UBE2C
Protein ubiquitination	1.56E‐05	6	UBE2V2, CDC34, UBR1, SKP1, UBE2C, UBA1
Protein polyubiquitination	2.29E‐05	5	UBE2V2, UBA52, CDC34, SKP1, UBC
DNA damage response, signal transduction by p53 class mediator resulting in cell cycle arrest	2.59E‐05	4	GTSE1, UBA52, CCNB1, UBC
Stress‐activated MAPK cascade	1.77E‐04	3	UBA52, SKP1, UBC
G2/M transition of mitotic cell cycle	2.75E‐04	4	UBA52, SKP1, UBC, CCNB1

**TABLE 5:** The KEGG pathway for the hub genes

Term	*P* value	Count	Genes
Oocyte meiosis	4.52E‐04	4	ESPL1, SKP1, CCNB1, MAD2L1
Cell cycle	6.25E‐04	4	ESPL1, SKP1, CCNB1, MAD2L1
Ubiquitin‐mediated proteolysis	.016	3	CDC34, SKP1, UBE2C, UBA1

## 4 DISCUSSION

Oocyte ageing is influenced by various biochemical factors. In this study, the scRNA‐seq analysis provided interesting data. Firstly, 481 DEGs were identified according to the established screening criteria, which consisted of 322 upregulated and 159 downregulated genes. Subsequently, functional enrichment analysis of DEGs involved in various biological processes, molecular functions, and KEGG pathways revealed that many of these genes appeared to play important roles in oocyte development.

A previous study had identified DEGs between old and young human oocytes via RNA‐seq.^8^ It was reported that the upregulated genes were significantly enriched in transcriptional activation, oxidative stress and immune function, while downregulated genes were mainly enriched in catalytic activity. TOP2B was selected as a key candidate gene, which was involved in catalytic enzyme activity. However, there are many molecular mechanisms involved in oocyte ageing, and it is important to explore the molecular mechanisms of oocyte ageing from different perspectives for studying fertility decline in older women. Compared to the previous study, the enrichment analysis of DEGs in this study was mainly focused on the functions of transcription, ubiquitination, cell cycle, oxidative phosphorylation, and oocyte meiosis. The following molecular functions were found to be highly enriched: protein binding, DNA binding, RNA binding, and chromatin binding, which were in accordance with the processes of oocyte meiosis and cell cycle progression. In the PPI network, one module was significantly enriched in the biological process of ubiquitination. Subsequently, 17 hub genes ware screened and UBE2C was found to be the top degree gene. One of the most enriched GO term of hub genes is “ubiquitination ‐related functions”; with “Cell cycle” being also an important term. The KEGG pathway of hub genes was mainly involved in oocyte meiosis, cell cycle, and ubiquitin‐mediated proteolysis. Therefore, we think that expression of most genes in oocytes was influenced by the ageing process. The results of this study suggest that the mechanisms of oocyte ageing can be elucidated by determining the exact roles that genes enriched in “ubiquitination” and “oxidative phosphorylation” play in oocyte meiosis.

### 4.1 Oxidative phosphorylation

Mitochondria is a key factor in the development of human oocytes and embryos. It is well‐established that mitochondria provides energy for transcription and translation during oocyte maturation.^14^ The development of mammalian oocytes depends almost entirely on oxidative phosphorylation to produce ATP.^15^ A study has shown that a slight decrease in ATP can irreversibly damage oocyte quality by disrupting the oocyte maturation process.^16^ Much evidence has shown that oxidative phosphorylation pathways play a crucial role in oxidative stress damage.^17^ Moreover, ATP production depends on the stability of mitochondrial membrane potential, and pathological stimulation can lead to a change of mitochondrial membrane potential, thereby increasing the reactive oxygen species (ROS) level.^18^ Our differential expression analysis showed that genes involved in oxidative phosphorylation pathways were downregulated, including COX6C (|log2FC| = 3.13), CYTB (|log2FC| = 2.06), ND4L (|log2FC| = 2.22), ND4 (|log2FC| = 2.19), ATP6V1C1 (|log2FC| = 1.85), ATP6V0D1 (|log2FC| = 2.25), and COX7A2L (|log2FC| = 1.36). Furthermore, the ATP synthesis‐coupled electron transport‐ and GDP binding‐related pathways were also downregulated, which is consistent with the decline in oxidative phosphorylation during oocyte ageing. Mitochondrial DNA codes for 13 subunits (ND1, ND2, ND3, ND4, ND4L, ND5, ND6, COXІ, COXⅡ, COXⅢ,Cytb, ATP6, ATP8) that are essential for respiration and oxidative phosphorylation.^19^ An increase in ROS level was thought to be associated with a decline in the quality of aged oocytes.^20^, ^21^ Once mtDNA is damaged, mutations easily occur in these 13 subunits, thus affecting the structure and function of the respiratory chain oxidative phosphorylase complex, resulting in increased electron leakage and production of high ROS levels.

### 4.2 Ubiquitination and ubiquitin‐mediated proteolysis

The ubiquitin‐proteasome system (UPS) involves two steps, ubiquitination and degradation. The oocyte maturation process is largely controlled by specific protein degradation mediated by the UPS, which has also been reported to be associated with mammalian fertilization and embryogenesis.^22^ The biological information mining data of each link showed that there were significant differences in the regulation of ubiquitination between young and old oocytes. Researchers have revealed that ubiquitination is involved in the regulation of various cellular biological events such as gene transcription, cell cycle process, DNA damage repair, apoptosis, ageing and fertility.^23^, ^24^, ^25^ Ubiquitination and ubiquitination‐related pathway including mitotic cell cycle, and meiosis are the most differentially enriched biological processes between old and young oocytes in our study. Cuervo et.al had shown that age‐related defect in ubiquitination involved less availability of free ubiquitin (Ub) and more Ub‐protein coupling, which led to inefficient protein recovery and recycling.^26^ Another study showed that the genes involved in UPS are downregulated in aged human oocytes.^27^ However, based on GO term and KEGG pathway analysis, 6 upregulated genes (ie UBE2C, UBC, GTSE1, CDC34, UBR1, UBA1) and 5 downregulated genes (ie UBA52, SKP1, CCNB1, MAD2L1, UBE2V2) are included in the ubiquitination‐related term.

The upregulated expression of hub genes such as CDC34, UBA1, UBE2C, and downregulated expression of hub gene SKP1 are enriched in ‘Ubiquitin‐mediated proteolysis’ pathway. Ubiquitin‐activated enzymes (E1) initiate the transfer of ubiquitin molecules to target proteins and are degraded by proteasomes. The gene transcript of UBA1, an E1, was upregulated by age. UBA1 is a key enzyme for ubiquitylation‐dependent signalling of both DNA double‐strand breaks (DSBs) and replication stress.^28^ If DNA damage is unrepaired, it can lead to an accumulation of genetic mutations related to ageing.^29^ CDC34 is the core of a family of enzymes, including the UBA1 and Skp‐Cullin‐F‐box (SCF) E3 ubiquitin ligase, which works in tandem to assemble lys48‐linked Ub chains on proteins to facilitate proteasomal degradation of target proteins.^30^, ^31^ UBE2C is an E2 enzyme, which showed the highest degree in the PPI network of hub genes, which is involved in mono‐ubiquitination or initial ubiquitination of substrates. The factors needed to maintain MII arrest are degraded by UBE2‐mediated mechanisms; high levels of UBE2C lead to premature activation of anaphase‐promoting complex, cyclosome (APC/C) and cytokinesis, which suggested that the threshold level of UBE2C may be reached in advance at the time of overexpression, leading to degradation of securin and cyclin B1, which ultimately leads to activation of ESPL1 and inactivation of CDK1. However, UBE2C depletion leads to inactivation of APC/C, which in turn results in improper spindle formation and SAC was unable to maintain silencing.^32^ Therefore, these findings demonstrated that dysregulation of UBE2C in aged oocytes may disrupt the meiotic process. Skp1 is a conserved subunit of the Skp1‐Cullin‐F‐box protein (SCF) E3 ubiquitin ligase. A lack of any protein may block the division of germ cells, so the SCF complex that regulates protein degradation plays a crucial role in this process. SCF ensures the separation of meiotic chromosomes by degrading meiotic recombination intermediates.^33^ However, whether abnormal expression of SKP1 will affect chromosome separation in human oocytes remains to be further verified. In addition, other ubiquitination‐related genes such as UBA52 is instrumental to the regulation of physiological ubiquitination and embryonic development, and modification/knockout of the UBA52 gene causes embryonic developmental arrest before implantation.^34^, ^35^ Thus, downregulated expression of UBA52 (1.4‐|log2FC|) may influence the formation of ubiquitin and oocyte development. UBC (3.9‐|log2FC|) is considered to be necessary for maintaining Ub homeostasis, which is mainly involved in the intracellular ubiquitin content and supply of extra‐Ub under stress.^36^ The correlation between the change of UBC expression and the decrease in free ubiquitin associated with aged oocytes needs to be further explored. In summary, changes in the expression levels of these ubiquitination‐related genes reflect the impairment of the balance of the ubiquitination system during oocyte ageing.

### 4.3 Oocyte meiosis and cell cycle

Oocyte maturation depends on the regulation of proteins that determine the correct assembly of meiotic spindles and precise separation of chromosomes. Chromosomal aneuploidy is caused by chromosomal separation errors during meiosis I and II which increases in aged oocytes.^37^, ^38^ In our study, three hub genes for ‘oocyte meiosis’ and ‘cell cycle’ SKP1, CCNB1, MAD2L1 were downregulated, but ESPL1 was upregulated. Due to the lack of de novo mRNA transcription during oocyte meiosis, the regulation of protein level, particularly the regulation of UPS, is more critical.^39^ SKP1 is not only involved in UPS but also in oocyte meiosis and cell cycle. Oocyte maturation is initiated by activation of maturation‐promoting factor (MPF), which is stimulated by cyclin B (CCNB) accumulation and chromosome segregation triggered by securin degradation.^40^ The UPS determines the availability of CCNB1 and thus regulates CDK1 activity.^32^ CCNB1 is one of the substrates of UBE2C ubiquitination. CCNB1 accumulation was observed to be decreased in UBE2C overexpressing oocytes cultured for 24h.^40^ However, some UBE2C overexpressing oocytes could extrude second polar bodies, and the synthesis of CCNB1 may be faster than its degradation; therefore, CCNB1 accumulates, CDK1 is activated, and MII arrest fails.^32^ Additionally, it has also been shown that in mouse oocytes, excessive CCNB1 protects CDK1 activity, which facilitates spindle formation and prevents aneuploidy.^41^ MAD2L1, also known as mitotic spindle assembly checkpoint protein MAD2, is related to the spindle assembly checkpoint (SAC), which regulates attachment of kinetochore microtubules.^42^ During mitosis and meiosis, SAC activity can affect the segregation of chromosomes.^43^ A previous study found that reduction of MAD2L1 levels caused an acceleration of meiosis I, but there was not enough time for the bipolar spindles to properly connect to functional kinetochores in MI oocytes; resulting in SAC being impaired, with consequent increase in the oocyte aneuploidy rate.^43^ RT‐PCR analysis of Mad2 transcripts during human meiosis has shown that MAD2L1 is degraded with age and may be a contributing factor to age‐related aneuploidy.^44^ These are consistent with our study that MAD2L1 is downregulated. Downregulation of MAD2L1 may increase the risk of premature chromosome separation.^45^ ESPL1 was reportedly upregulated in aged oocytes, which can lead to premature separation of chromatids.^46^ The genes involved in spindle formation and chromosome segregation in this study are influenced by age and are closely associated with ubiquitination, which may partly explain the pathogenesis of poor oocyte quality in older female patients.

In conclusion, our bioinformatics analysis revealed various DEGs and signalling pathways involved in oocyte ageing. The identified hub genes will be the focus of our future research. Although the results need to be further validated by RT‐PCR, cell function studies, and animal experiments, these provide cues for future research directions on the molecular mechanisms of oocyte ageing. Our data strongly suggest that oocyte meiosis, cell cycle, and UPS pathway play an important role in oocyte ageing.

## CONFLICT OF INTEREST

All authors declare that the research was conducted in the absence of any commercial or financial relationships that could be construed as a potential conflict of interest.

## AUTHOR CONTRIBUTION

**Lihua Yuan:** Conceptualization (equal); Data curation (lead); Formal analysis (lead); Methodology (lead); Software (lead); Writing‐original draft (lead); Writing‐review & editing (equal). **Ping Yin:** Conceptualization (equal); Data curation (equal); Formal analysis (equal); Methodology (equal); Writing‐original draft (supporting). **Hua Yan:** Conceptualization (equal); Supervision (equal). **Xiufang Zhong:** Conceptualization (equal); Data curation (equal). **Chunxia Ren:** Methodology (supporting). **Kai Li:** Resources (equal); Writing‐original draft (supporting). **Boon Chin** **Heng:** Validation (supporting); Visualization (lead). **Wuwen Zhang:** Conceptualization (equal); Funding acquisition (equal); Supervision (equal). **Guoqing Tong:** Conceptualization (lead); Funding acquisition (lead); Project administration (lead); Supervision (lead); Validation (lead).

## Supporting information

Fig S1

Fig S2

Table S1

Table S2

## Data Availability

The data that support the findings of this study are openly available in GEO database at https://www.ncbi.nlm.nih.gov/geo/, reference number GSE155179.
