# Supplementary material for: Single‐cell transcriptome analysis of human oocyte ageing
Source: J Cell Mol Med. 2021 May 26;25(13):6289–303. doi: 10.1111/jcmm.16594 (PMC8256362; doi:10.1111/jcmm.16594)
Supplement: Supplementary file 1 — Fig S1 [file JCMM-25-6289-s002.docx]

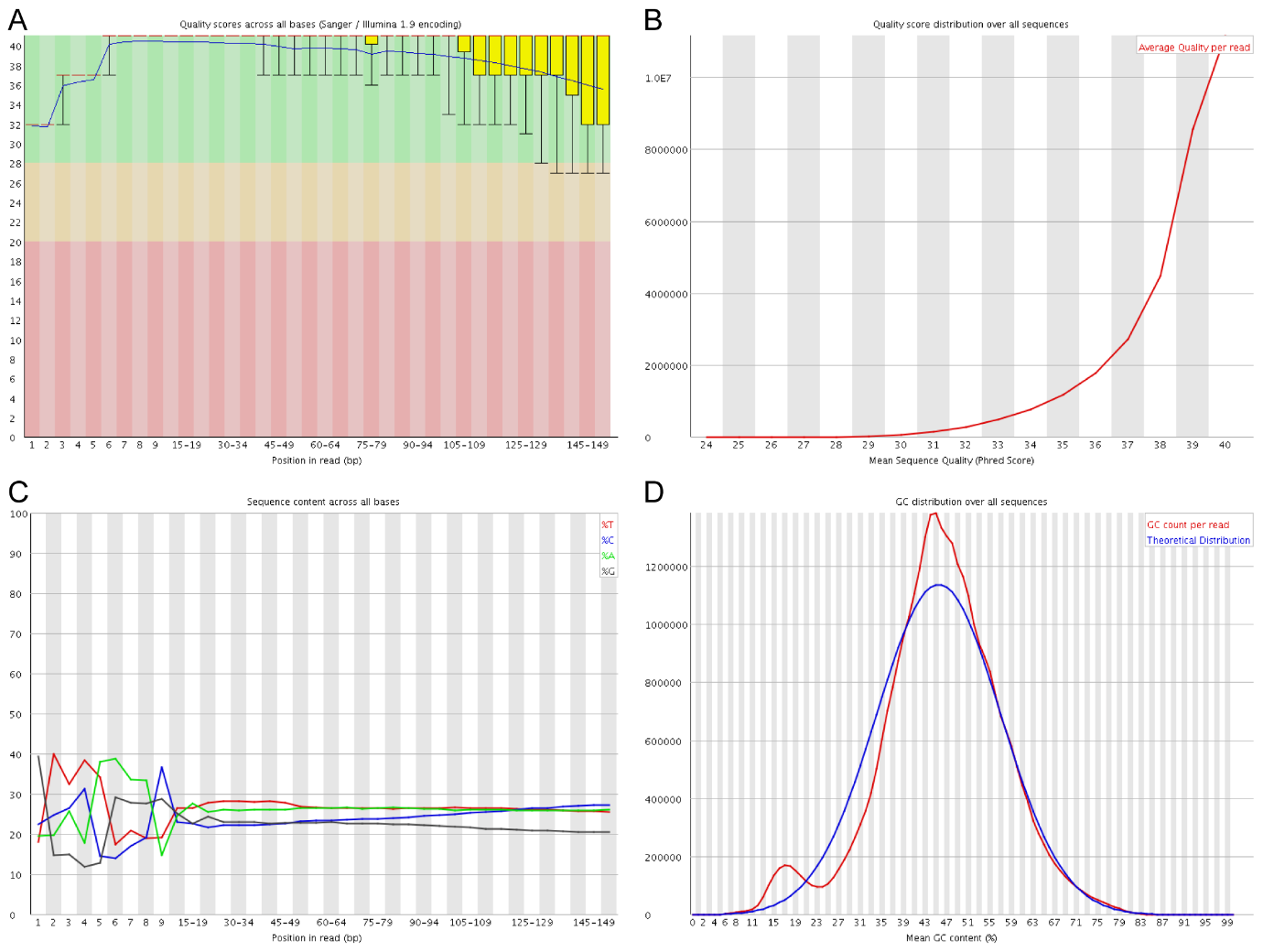


**Figure S1.** Quality filtration and quality control analysis by FastQC. (A) Box plot of sequence quality distribution; The abscissa is the base position of reads (5’->3’), and the ordinate is the base mass statistics of all reads at this site; The average quality of filtered data from this sequencing process is high, and it is basically distributed in the green area (Q >28). (B) Base mass distribution; The abscissa is the average mass of reads and the ordinate is the number of reads; The quality of most reads was distributed around 40, indicating that the average quality of overall reads was very high. (C) Base distribution.The abscissa is the base position of reads, and the ordinate is the percentage of various bases of all reads at this site. (D) GC content distribution diagram. The abscissa is the GC ratio, and the ordinate is the reads number; The red curve represents the measured value, while the blue curve represents the theoretical value; The shape is close to normal but deviates from the theoretical distribution, which suggests that there may be a systematic bias.
