# Supplementary material for: Single‐cell transcriptome analysis of human oocyte ageing
Source: J Cell Mol Med. 2021 May 26;25(13):6289–303. doi: 10.1111/jcmm.16594 (PMC8256362; doi:10.1111/jcmm.16594)
Supplement: Supplementary file 2 — Fig S2 [file JCMM-25-6289-s001.docx]

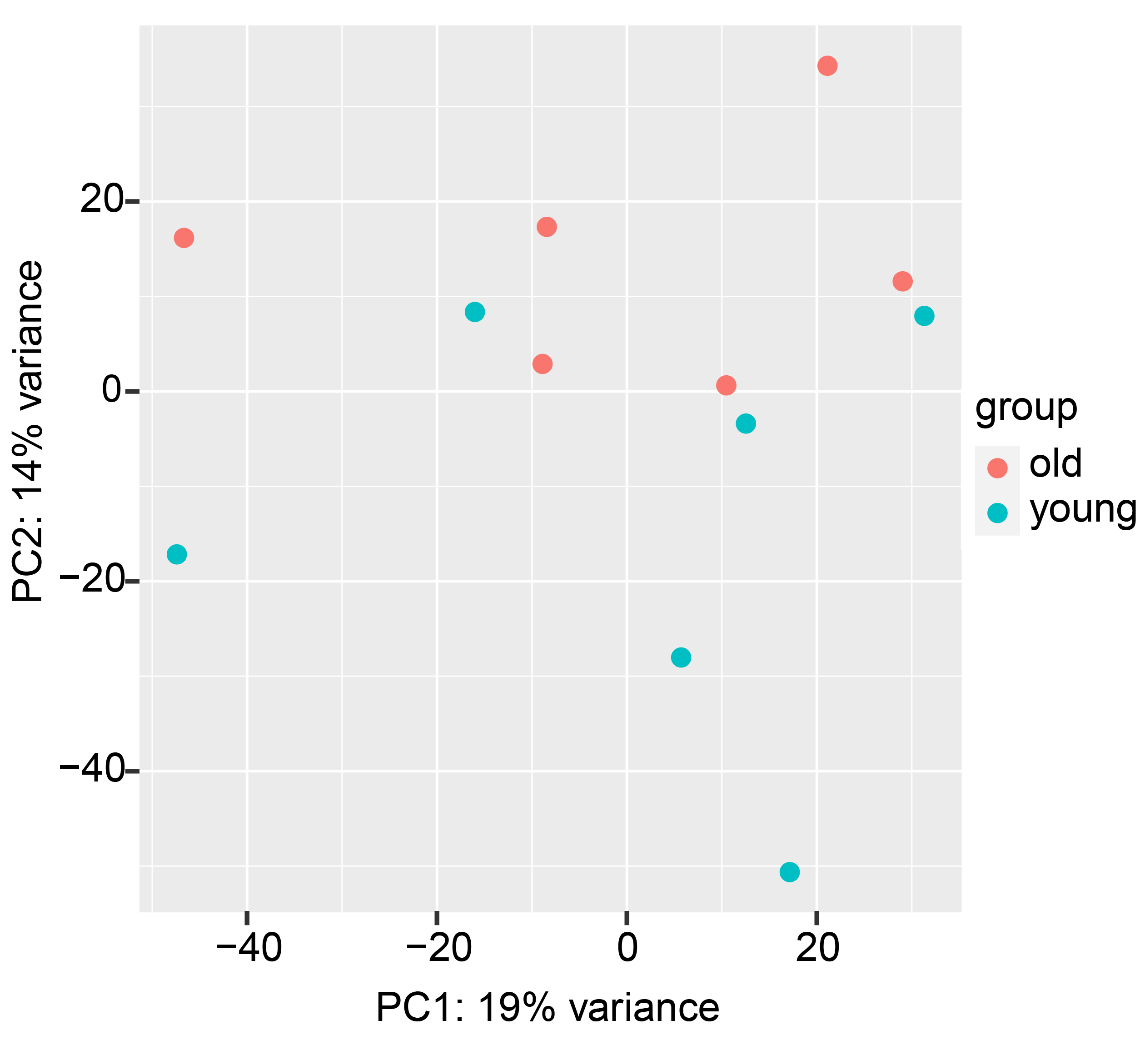


**Figure S2.** Principal Component Analysis (PCA) Score Chart in 6 old and 6 young oocyte samples. The results showed that three of the younger oocyte samples did not display any tendency to separate from the older samples.
