# Supplementary material for: Single‐cell transcriptome analysis of human oocyte ageing
Source: J Cell Mol Med. 2021 May 26;25(13):6289–303. doi: 10.1111/jcmm.16594 (PMC8256362; doi:10.1111/jcmm.16594)
Supplement: Supplementary file 3 — Table S1 [file JCMM-25-6289-s003.docx]

**Table S1** Patient baseline characteristics and clinical laboratory outcomes in the two age groups for analyzing gene expression in oocytes.

|  | **Younger** | **Older** |
| --- | --- | --- |
| Number of donated oocytes | 3 | 6 |
| Age (years) | 25.67±2.08 | 42.67±2.25* |
| BMI (Kg/m^2^) | 21.51±1.56 | 22.39±2.10 |
| bFSH (IU/L) | 4.07±0.79 | 11.93±1.54* |
| bLH | 4.06±1.38 | 8.44±2.33* |
| bE2 | 35.00±2.65 | 56.83±26.95 |
| Infertility (years) | 3.00±1.00 | 3.17±1.17 |
| No. oocytes retrieved | 12.67±3.06 | 3.67±1.21* |
| MⅡoocyte transition rate | 86.8% (33/38) | 77.3% (17/22) |
| Normal fertilization rate | 87.9% (29/33) | 64.7% (11/17) |
| Available embryo rate | 88.5% (23/26) | 60.00% (6/10) |

bE2, basal estradiol; bFSH, basal follicle-stimulating hormone; bLH, basal luteinising hormone; BMI, body mass index; Data are presented as mean±SD and frequency (%); *P < 0.05 is considered significantly different.
