# Supplementary material for: Single‐cell transcriptome analysis of human oocyte ageing
Source: J Cell Mol Med. 2021 May 26;25(13):6289–303. doi: 10.1111/jcmm.16594 (PMC8256362; doi:10.1111/jcmm.16594)
Supplement: Supplementary file 4 — Table S2 [file JCMM-25-6289-s004.docx]

**Table S2** Patient baseline characteristic and clinical laboratory outcomes in the younger group of 3 included and 3 excluded samples.

|  | **Younger (included)** | **Younger (excluded)** |
| --- | --- | --- |
| Number of donated oocytes | 3 | 3 |
| Age (years) | 25.67±2.08 | 28.00±1.00 |
| BMI (Kg/m^2^) | 21.51±1.56 | 21.64±1.31 |
| bFSH(IU/L) | 4.07±0.79 | 6.71±0.73* |
| bLH | 4.06±1.38 | 5.56±0.82 |
| bE2 | 35.00±2.65 | 45.67±18.34 |
| Infertility (years) | 3.00±1.00 | 4.33±1.53 |
| No. oocytes retrieved | 12.67±3.06 | 8.33±1.53 |
| MⅡoocyte transition rate | 86.8% (33/38) | 64.00% (16/25)* |
| Normal fertilization rate | 87.9% (29/33) | 81.3% (13/16) |
| Available embryo rate | 88.5% (23/26) | 53.8% (7/13)* |

bE2, basal estradiol; bFSH, basal follicle-stimulating hormone; bLH, basal luteinising hormone; BMI, body mass index; Data are presented as mean±SD and frequency (%); *P < 0.05 is considered significantly different.
